# Abnormal breathing patterns and hyperventilation are common in patients with chronic fatigue syndrome during exercise

**DOI:** 10.3389/fmed.2025.1669036

**Published:** 2025-11-10

**Authors:** Donna M. Mancini, Danielle L. Brunjes, Dane Cook, Tiffany Soto, Michelle Blate, Patrick Quan, Tadahiro Yamazaki, Anna Norweg, Benjamin H. Natelson

**Affiliations:** 1 Department of Cardiology, Icahn School of Medicine at Mount Sinai, New York, NY, United States; 2 Department of Population Health Science and Policy, Icahn School of Medicine at Mount Sinai, New York, NY, United States; 3 Department of Kinesiology, University of Wisconsin, Madison, WI, United States; 4 Department of Neurology, Icahn School of Medicine at Mount Sinai, New York, NY, United States

**Keywords:** chronic fatigue syndrome, exercise, hyperventilation, dysfunctional breathing disorder, cardiopulmonary exercise test

## Abstract

**Introduction:**

Patients with myalgic encephalomyelitis/chronic fatigue syndrome (ME/CFS) experience symptoms of fatigue, dyspnea, mental fog, and worsening fatigue after physical or mental efforts. Some of these patients have been found to hyperventilate. In long COVID patients, many of whom also have ME/CFS, dysfunctional breathing (DB) has been described. Whether patients with ME/CFS, independent of COVID-19, experience dysfunctional breathing is unknown, as well as how it may relate to hyperventilation.

**Methods:**

We performed serial 2-day cardiopulmonary exercise testing (CPET) in 57 patients with ME/CFS and 25 age- and activity-matched control participants. Peak oxygen consumption (VO_2_), ventilatory efficiency slope (VE/VCO_2_), O_2_ saturation, end-tidal CO_2_ (PetCO_2_), heart rate, and mean arterial blood pressure were measured in all patients during upright incremental bicycle exercise. Ventilatory patterns were reviewed using minute ventilation (VE) versus time, respiratory rate, and tidal volume versus minute ventilation graphs. Chronic hyperventilation (HV) was defined as a PETCO_2_ of <34 mm Hg that persisted during low-intensity exercise. Dysfunctional breathing was characterized by a 15% increase in oscillations in minute ventilation during at least 60% of the exercise duration or by a scatterplot pattern of respiratory rate and tidal volume plotted versus minute ventilation.

**Results:**

The patients with ME/CFS had an average age of 38.6 ± 9.6 years, and a mean body mass index (BMI) of 24.1 ± 3.4, which was comparable to the sedentary controls. All participants performed maximal exercise, achieving a respiratory exchange ratio (RER) of >1.05. For the patients with ME/CFS, peak VO_2_ averaged 22.3 ± 5.3 mL/kg/min, which was 79 ± 20% of predicted and comparable to that observed in the sedentary controls (23.4 ± 4.6 mL/kg/min; 81 ± 12%; p = NS). A total of 24 patients with ME/CFS (42.1%) met the criteria for dysfunctional breathing compared to four sedentary controls (16%) (*p* < 0.02). In total, 18 patients with ME/CFS (32%) had hyperventilation compared to one sedentary control participant (4%) (*p* < 0.01), and nine patients with ME/CFS had both hyperventilation and dysfunctional breathing, whereas no sedentary participant exhibited both. The patients with ME/CFS and hyperventilation had significantly higher VE/VCO_2_ ratios (HV+: 34.7 ± 7.2; HV−: 28.1 ± 3.8; *p* < 0.001). A total of 15 of 18 patients with hyperventilation (83%) had either elevated VE /VCO_2_ ratios (*n* = 15) or dysfunctional breathing (*n* = 9) compared to 44% (*n* = 17) of the 40 non-hyperventilators (*p* < 0.01).

**Conclusion:**

Dysfunctional breathing and hyperventilation are common in patients with ME/CFS and could present a new therapeutic target for these patients.

## Introduction

Myalgic encephalomyelitis/chronic fatigue syndrome (ME/CFS) is a medically unexplained illness characterized by at least 6 months of unexplained fatigue, severe enough to cause a substantial reduction in activity. In addition to fatigue, patients commonly report symptoms including dyspnea, mental fog, and worsening fatigue following minor physical or mental efforts [post-exertional malaise (PEM)] (1). PEM is common in ME/CFS and is thought to be a key characteristic of this disease.

Dyspnea is not an infrequent problem (2), and both hyperventilation (3, 4) and inefficient ventilation (5) have been reported in ME/CFS cases. Chronic hyperventilation (HV) syndrome is associated with substantial fatigue in up to 64% of patients (3) and is assessed using methods similar to those for patients with ME/CFS (3, 4). In our prior study of patients with ME/CFS, 14 of 63 patients were hypocapnic at rest [22%] (6). Abnormal patterns of ventilation can be associated with dyspnea.

Dysfunctional breathing (DB) is characterized by rapid, erratic respirations with significant variability in respiratory rate and tidal volume during exercise, in contrast to the normal exercise ventilatory response, which follows a pattern of a linear rise in respiratory rate and an early rapid increase in tidal volume that plateaus as peak exercise nears (7). Dysfunctional breathing has most commonly been described in patients with asthma. It can result from functional and non-functional factors, be associated with many non-specific symptoms including dyspnea and fatigue, and occur independently or together with hyperventilation (8). A proposed classification of dysfunctional breathing patterns includes periodic deep sighing, thoracic dominant breathing, forced abdominal expiration, and thoraco-abdominal asynchrony (8). Diagnostic techniques include questionnaires such as the Nijmegen Questionnaire, capnography, breathing pattern assessment using plethysmography, the breath-hold test, and cardiopulmonary exercise testing (CPET) (7, 8).

Previously, we investigated unexplained dyspnea in patients with post-acute sequelae of COVID-19 (PASC) (9) and observed that nearly half of these patients met the criteria for ME/CFS. The development of ME/CFS has frequently been reported following viral infections. Using CPET, we also described a high incidence of ventilatory inefficiency and dysfunctional breathing in PASC (9). Estimates of dysfunctional breathing in the general population are around 9% (10, 11).

Both hyperventilation and dysfunctional breathing can result in a constellation of symptoms, such as dyspnea, dizziness, problems with attention and concentration, and fatigue, which overlap with those experienced by patients with ME/CFS (12, 13).

Several investigators have used two sequential CPET to infer PEM in patients with ME/CFS (14–18). Metabolic carts used for this testing measure end-tidal CO_2_, breathing frequency, and minute ventilation (VE). If, following the second test, reduced maximal oxygen consumption (VO_2_max) and/or an earlier onset of the anaerobic threshold were found, researchers concluded that these results reflect PEM and represent a manifestation of disability (14–20).

Although the breathing pattern during exercise in patients with ME/CFS is not well described, a recent study reported an elevated VE/VCO_2_ ratio consistent with excessive ventilation (5). This excessive ventilation exceeds that needed to support exercise (21) and increases respiratory muscle work, which may affect perceived dyspnea and fatigue.

The purpose of this study was to use CPET to determine the frequency and relation of hyperventilation and dysfunctional breathing in patients with ME/CFS whose disease was not triggered by COVID-19.

## Methods

### Patient population

The patients with ME/CFS were between the ages of 25 and 60 years and met the 2015 IOM case definition (22). Therefore, they all reported a new onset of fatigue, lasting at least 6 months and causing at least a substantial decrease in activity. In addition, they reported experiencing at least a substantial burden (≥3 on a 0-to-5 visual analog scale) of some of the following symptoms in the month prior to the study: unrefreshing sleep, post-exertional malaise, problems with attention/concentration, or orthostatic sensitivity. A similar scale was used to assess dyspnea. Patients with a medical cause for their fatigue, those taking medications that would dampen cardiac response to exercise, or those with any of the following psychiatric conditions were excluded: psychotic illness, bipolar disorder, history of anorexia or bulimia within 5 years of intake, history of alcohol or drug abuse within 2 years of intake, and current major depressive disorder. Patients with Ehlers–Danlos syndrome were also excluded.

The normal controls were sedentary individuals (*N* = 25) who did not exercise regularly, had no known medical problems, and were on no medication other than birth control pills. None of these participants had a recent viral infection. These participants were selected to match the patients with ME/CFS based on age, gender, body mass index (BMI), and activity using the Godin Leisure-Time Exercise Questionnaire (10). This scale grades individuals on the following levels of activity using arbitrary units: active (≥24 Units), moderately active (14–23 units), or sedentary (<14 Units) (23).

The study was approved by the Institutional Research Board of the Icahn School of Medicine at Mount Sinai. All patients provided informed consent.

### Cardiopulmonary exercise testing

The patients reported to the exercise laboratory in the fasting state. Medications were continued. The patients were connected to an EKG, pulse oximeter, and BP cuff and seated on a bicycle ergometer (Lode, Groningen, Netherlands). End-tidal CO_2_ < 34 mm Hg was used to define hypocapnia and hyperventilation. To differentiate acute from persistent hyperventilation, normalization of PaCO_2_ by the end of the 25-W workload was considered consistent with acute hyperventilation caused by the stress of undergoing CPET, which requires the use of a mouthpiece with nasal occlusion.

Using a disposable mouthpiece, the patients breathed into a metabolic cart (Med Graphics Ultima O_2_). Resting data were collected for 2 min, followed by incremental bicycle exercise starting at 0 W and increasing by 25 W every 2 min until exhaustion. VO_2_ consumption, VCO_2_ production, respiratory frequency, minute ventilation, end-tidal CO_2_, and O_2_ were recorded continuously. O_2_ saturation and estimated arterial PCO_2_ were also recorded. Blood pressure and perceived fatigue, assessed using the Borg scale, were recorded at each workload and at peak exercise. The reason for terminating exercise was recorded. The cardiopulmonary exercise test was repeated after 24 h. The results of the first exercise test are reported. However, only the breathing patterns from the second test are included to analyze the reproducibility of these data.

Peak VO_2_ was defined as the highest 30-s average of oxygen consumption and was normalized by the predicted VO_2_ (Wasserman’s equation) to calculate a percentage of the predicted value. The ventilatory threshold was identified as the point at which the ventilatory equivalent for O_2_ was minimal, followed by a progressive increase. The ventilation (VE) to carbon dioxide production (VCO_2_) slope was assessed using the correlation between VE and VCO_2_ throughout exercise. A normal VE/VCO2 slope is <30 (24). Hypocapnia was defined as a resting end-tidal CO_2_ (P_ET_CO_2_) below the lower limit of normal (<34 mm Hg). Persistent hyperventilation (HV) was defined as hypocapnia at rest that persisted throughout low-level exercise, that is, up to 25 W. A maximal test was defined if either of the following criteria was met: respiratory exchange ratio (RER) ≥ 1.05 or heart rate >85% of predicted (24).

There are no strict criteria for the identification of dysfunctional breathing. Identification is based on pattern recognition (7). We reviewed graphs of minute ventilation (VE) versus time, as well as respiratory rate (RR) and tidal volume (VT) versus VE (ml/min). For the VE versus time graph, we applied the American Heart Association’s definition of exercise oscillatory ventilation—that is, cyclic ventilation that persists for at least 60% of the exercise test with an amplitude 15% or more above resting values—to identify significant breathing abnormalities, specifically dysfunctional breathing (25).

For the plots of tidal volume and respiratory rate versus minute ventilation, a normal pattern generally shows an early rapid rise in tidal volume that plateaus, accompanied by an initial slow, then progressively faster, rise in respiratory rate—yielding a football-shaped plot. With dysfunctional breathing, there is marked variability in respiratory rate and tidal volume throughout most of the exercise, yielding a scatterplot graph. Dysfunctional breathing was identified if a participant’s data showed either oscillatory ventilation in the VE versus time plot or a scatterplot pattern in the VT versus VE and RR graphs.

## Data and statistical analysis

All continuous variables were presented as mean ± standard deviation. Variables were compared between the groups using non-paired *t*-tests and within the groups using paired *t*-tests, assuming equal variance. The results were considered significant if the two-tailed *p*-value was <0.05. The analysis was performed using SPSS (version 31). Categorical variables were summarized as frequencies and percentages. The results involving multiple groups were compared using one-way ANOVA. The relationship between breathing condition and severity of symptoms was assessed using independent *t*-tests.

## Results

The study included 57 patients with ME/CFS (F:M = 46:11) with an average age of 38.6 ± 9.6 years and a mean BMI of 24.1 ± 3.4 and 25 sedentary controls (F:M = 20:5) with an average age of 38.2 ± 9.9 years and a mean BMI of 24.2 ± 3.4. There was no significant difference in the Godin Leisure-Time Exercise Questionnaire between the two groups (ME/CFS: 27.7 ± 35.5; Controls: 13.5 ± 14.4; *p* = 0.22), indicating that the healthy controls were as sedentary as the patients. All patients with ME/CFS and sedentary controls achieved an RER > 1.05, with a mean Borg scale rating of 18 at peak exercise (Table 1). In the patients with ME/CFS, peak VO_2_ averaged 22.4 ± 5.4 mL/kg/min, which was 79 ± 20% of predicted. The results from the 25 sedentary controls were comparable, although maximal HR was higher in the control group (*p* < 0.05) and perceived exertion at end exercise was significantly lower (*p* < 0.001).

**Table 1:** Cardiopulmonary exercise test results.

Parameter	ME/CFS			Sedentary control		
	Rest	VT	Peak	Rest	VT	Peak
HR (bpm)	81 ± 14	111 ± 17	157 ± 19^	83 ± 14	116 ± 14	166 ± 11^*
MAP (mmHg)	87.6 ± 8.5	93.2 ± 9.6	102.9 ± 12^	89 ± 6	95.6 ± 6.7	104 ± 7^
Respiratory rate	15.1 ± 4.6		32.1 ± 7.1	14.5 ± 4.3		34.8 ± 7.8
VO_2_ (ml/kg/min)	4.2 ± 0.84	12.6 ± 3.4	22.3 ± 5.3^	4.0 ± 0.8	13.4 ± 4.0	23.4 ± 4.6^
RER	0.91 ± 0.12	0.89 ± 0.09	1.19 ± 0.08^	0.97 ± 0.21	0.89 ± 0.1	1.21 ± 0.1^
P_et_CO_2_ (mm Hg)	34.2 ± 5.0	40.6 ± 4.4	37.4 ± 4.9^	34.3 ± 6.0	42.2 ± 4.1	39.6 ± 4.5^
VE/VCO_2_ slope			29.2 ± 5.9			27.2 ± 3.7
Borg		12.5 ± 2.4	18.0 ± 1.5		9.8 ± 2.7*	16.2 ± 2.8*

*p < 0.05 ME/CFS versus sedentary controls; ^*p* < 0.05 rest versus exercise.

HR, heart rate; MAP, mean arterial pressure; RER, respiratory exchange ratio; VO_2_, Oxygen Consumption; P_ET_CO_2_, end-tidal carbon dioxide pressure; Borg, visual analog scale; VT, anaerobic threshold.

**Figure 1:**
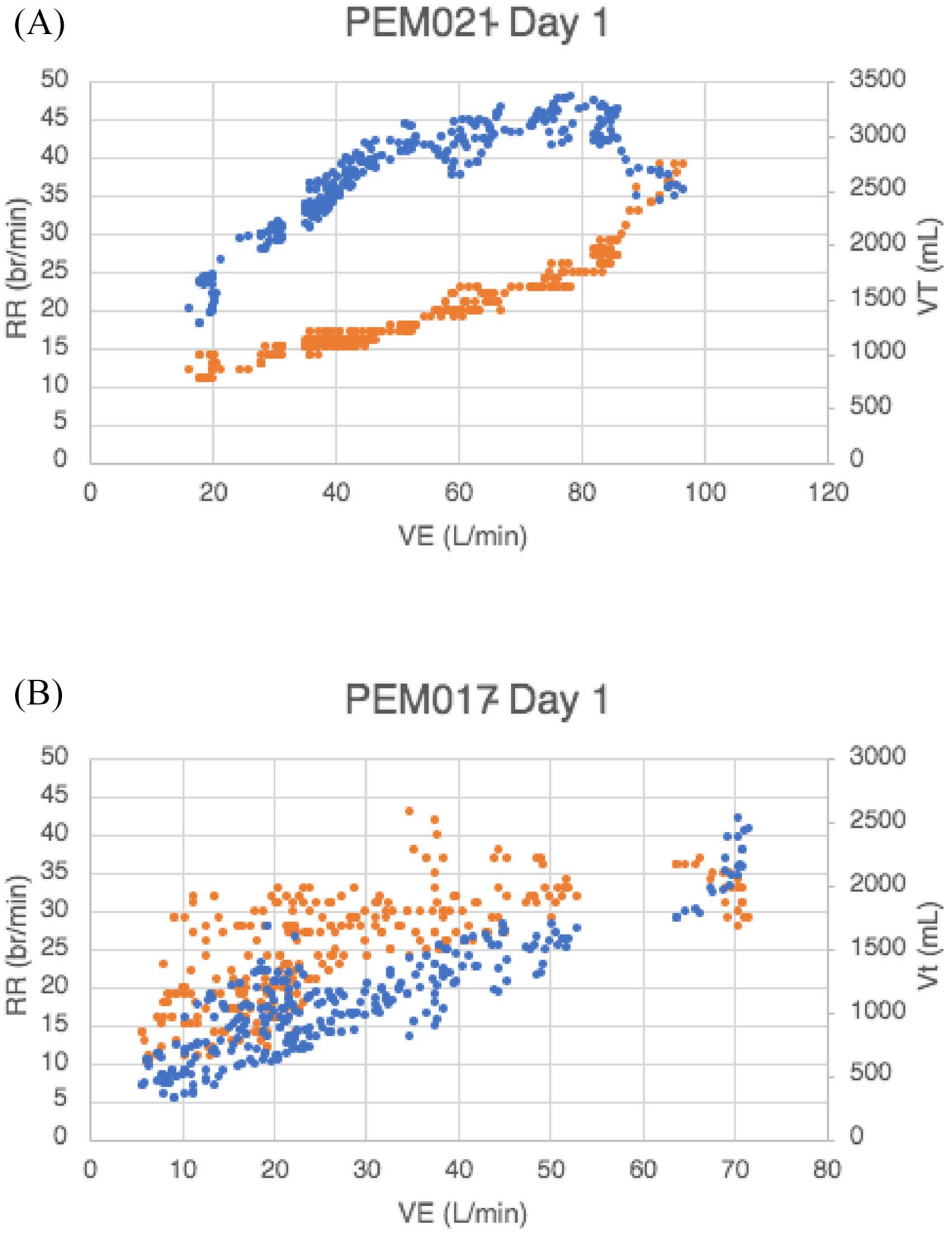
**(A)** Normal and **(B)** dysfunctional respiratory patterns in two different ME/CFS cases, with respiratory rate (RR) on the left y-axis and tidal volume (VT) on the right y-axis plotted against minute ventilation (VE) on the x-axis. (RR = respiratory rate, orange dots; VT tidal volume, blue dots) (see text).

*Dysfunctional Breathing (DB)* (see Table 2): The incidence of dysfunctional breathing was significantly greater in the patients with ME/CFS than the sedentary controls (*p* < 0.05). Figure 1A shows an example of a patient with ME/CFS demonstrating a normal ventilatory response plotted as respiratory frequency and tidal volume versus minute ventilation. The rapid rise in tidal volume reaching a plateau and the linear rise in respiratory rate led to an oblong configuration. In contrast, Figure 1B illustrates an erratic, disorganized breathing pattern, characterized by a rapid rise in respiratory rate and small tidal volumes, giving the appearance of a scatterplot.

**Table 2:** Patients with chronic fatigue versus sedentary controls stratified by the presence or absence of hyperventilation.

ME/CFS	All	No HV	HV
Number	57	39	18
Rest PetCO_2_	34 ± 5.1	36.3	28.1 ± 4.0*
25 W PetCO_2_	37.2 ± 4.8	40.5	31.8 ± 3.4*
RER @rest	0.91 ± 0.12	0.87 ± 0.09	1.10 ± 0.19*
VE/VCO_2_ slope	29.2 ± 5.9	28.1 ± 3.8	34.7 ± 7.2*
VE/VCO_2_ @ VT		27.6 ± 3.4	32.2 ± 3.0*
VE/VCO_2_ @ Peak exercise		29.2 ± 3.8	35.8 ± 3.7*
DB by VE/VT/RR	16	10	6
DB by oscillations	19	10	9
DB by either criteria	24	15	9
%DB	42.1^	38.5	50
Sedentary controls			
Number	25	24	1
Rest PetCO_2_	34.0 ± 5.5	34.3 ± 5.4	27
25 W PetCO_2_	38.3 ± 4.4	38.9 ± 3.5	26
VE/VCO_2_ slope	27.7 ± 3.4	27.8 ± 3.5	26
DB by VE/VT/RR	2	1	1
DB by oscillations	4	3	1
DB by either criteria	4	3	1
%DB	16^	12.5	100

^*p* < 0.05, sedentary controls versus ME/CFS cases; *p < 0.05, no HV versus HV.

HV, persistent hyperventilation; P_ET_CO_2_, end-tidal pressure of carbon dioxide; RER, respiratory exchange ratio; VE/VCO_2_, ratio of minute ventilation (VE) to carbon dioxide production; VT, anaerobic threshold; DB, dysfunctional breathing; DF by VE/VT/RR, the number of abnormal graphs (i.e., scatterplots); DB by oscillations, defined by the American Heart Association criteria.

A total of 24 patients showed evidence of dysfunctional breathing (42.1%). In addition, 17 patients showed a scatterplot pattern (29.8%) on the RR-VT-VE graph (Figure 1), 19 patients had oscillatory ventilation, and 12 patients had both. The breathing patterns observed during the Day 1 exercise test were reproducible on Day 2 in 93% of the patients. Among those with changes, two patients developed scatterplot-type breathing patterns, while two patients with scatterplot-type breathing patterns on Day 1 showed a normal pattern on Day 2.

A total of four sedentary controls (16%) met the criteria for dysfunctional breathing using either measure. Furthermore, two controls showed scatterplot-type breathing patterns on the VE-VT-RR graph (8%), four controls had oscillatory ventilation, and two controls met both criteria. The VE versus time graphs were reproducible on Day 2 in all controls.

### Hyperventilation (HV)

On Day 1, 23 patients (40.7%) had a PETCO_2_ of < 34 at rest, 12 patients normalized their P_ET_CO_2_ at the 25-W workload, consistent with acute hyperventilation, and 11 patients continued to show hypocapnia at the 25-W workload, consistent with persistent hypocapnia.

**Figure 2:**
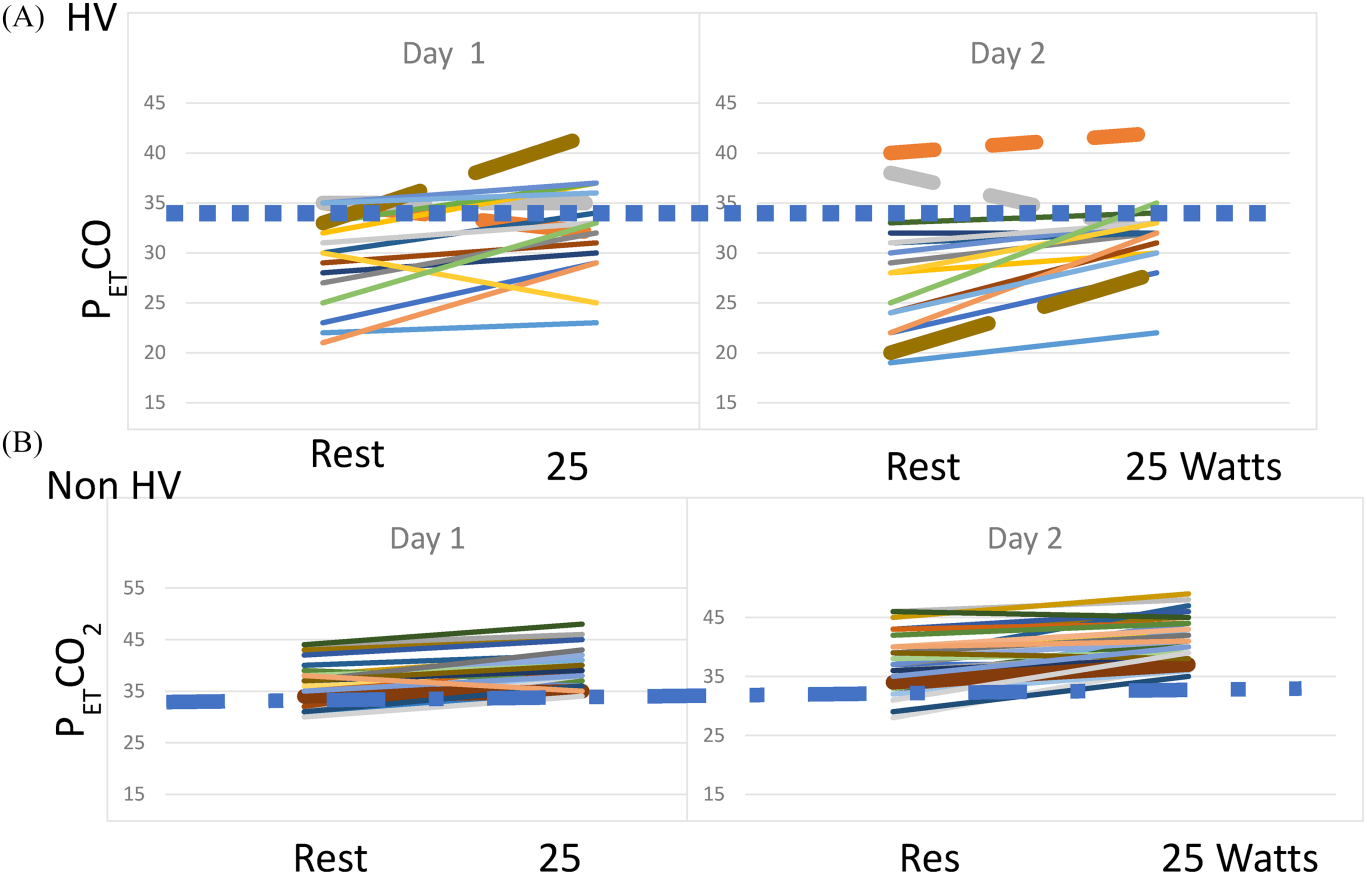
**(A)** P_ET_CO_2_ at rest and 25 W on Day 1 and Day 2 of CPET in the patients with ME/CFS and persistent hyperventilation. The dashed line indicates a P_ET_CO_2_ of 34 mm Hg. **(B)** P_ET_CO_2_ at rest and 25 W on Day 1 and Day 2 of CPET in the patients with ME/CFS and no hyperventilation. The dashed line indicates a P_ET_CO_2_ of 34 mm Hg.

On repeat testing, every patient showing hyperventilation was hypocapnic at rest on Day 2, with only one reaching borderline normocapnia at the end of the 25-W workload (PetCO_2_ = 35). Of the 12 patients with acute resting hypocapnia on Day 1, only one was normocapnic on Day 2, while four of the 10 patients with acute resting hypocapnia remained hypocapnic at 25 W. Among the 34 normocapnic patients on Day 1, four patients exhibited hypocapnia at rest on Day 2, with two continuing to show hypocapnia throughout the 25-W workload. Only one patient with normocapnia developed hypocapnia during low-level exercise. Over the course of the 2 days of testing, 18 ME/CFS cases (31%) showed evidence of hyperventilation during low-level exercise. Figure 2 shows the P_ET_ CO_2_ levels at rest and at 25 W of exercise in the patients with ME/CFS and persistent hyperventilation and in those who did not hyperventilate. The dotted line in both graphs indicates a P_ET_CO_2_ of 34 mm Hg.

Of note, P_ET_CO_2_ was significantly lower at rest, anaerobic threshold, and maximal exercise in the patients with ME/CFS who hyperventilated compared to those who did not (*p* < 0.001 for all three timepoints. Rest: HV: 28.3 ± 5.7, Non-HV: 36.9 ± 4.5; AT: HV: 36.2 ± 4.0, Non-HV: 42.7 ± 4.8; Max: HV: 32.1 ± 3.4, Non-HV: 39.8 ± 4.8 mm Hg). The resting RER was significantly higher in the HV group than in the non-HV group (non-HV: 0.87 ± 0.09; HV: 1.10 ± 0.19; *p* < 0.01).

For the sedentary controls, on Day 1 of testing, 11 individuals showed resting hypocapnia, but only one control had hypocapnia at 25 W (4%). On Day 2 of CPET, only six sedentary controls (24%) showed resting hypocapnia, and all had normal PetCO_2_ levels at 25 W. The decrease in the number of healthy controls showing acute resting hypocapnia suggests that these individuals habituated to the testing procedures.

Therefore, across the 2 days of testing, 18 of the ME/CSF patients showed evidence of hyperventilation (31.6%) during exercise. Only one sedentary control showed evidence of hyperventilation (4%); the difference between the groups was significant (*p* < 0.01).

Regarding ventilatory efficiency, the VE/VCO_2_ ratios on Days 1 and 2 of testing were mostly comparable; however, on Day 2, four additional ME/CSF cases exhibited a VE/VCO_2_ ratio of >30, while five ME/CSF cases showed a VE/VCO_2_ ratio of <30. For the sedentary controls, there was no change in the number of those above or below the VE/CO_2_ cut-off of 30, with elevated VE/VCO_2_ ratios seen predominantly in the acute hyperventilators (*n* = 6) (24%). The patients with ME/CFS and hyperventilation had statistically significantly higher VE/VCO_2_ ratios.

### Overlap of hyperventilation and dysfunctional breathing

A total of 33 of the 57 patients with ME/CFS (58%) had either HV or DB compared to four of the 25 normal controls (16%) (*p* = 0.001), demonstrating a greater incidence of these respiratory abnormalities in the ME/CFS group.

In the ME/CFS group with HV, nine (50%) had dysfunctional breathing and 11 had elevated VE/VCO_2_ slopes (61%). Among the remaining 39 patients, 16 (40%) showed evidence of dysfunctional breathing and seven (20%) had an elevated VE/VCO_2_ ratio. Therefore, the incidence of dysfunctional breathing was significantly higher in both hyperventilating and non-hyperventilating patients with ME/CFS.

**Figure 3:**
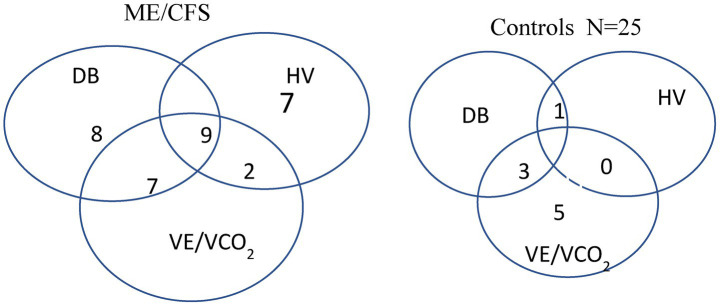
Venn diagrams showing the overlap of dysfunctional breathing, persistent hyperventilation, and elevated VE/VCO_2_ slope in the patients with ME/CFS and sedentary controls.

The VE/VCO_2_ ratio was elevated in 19 patients with ME/CFS and overlapped with chronic hyperventilation or dysfunctional breathing (Figure 3). In contrast, the VE/VCO_2_ slope was increased in seven sedentary controls, all with low resting PetCO_2_. Only one of them had dysfunctional breathing, and none had chronic hyperventilation (Figure 3).

Concerning the different breathing patterns and symptoms, there were no significant differences between HV alone or DB alone compared to normal breathing. However, differences were found for those with both DB and HV compared to normal breathing [*t* = 2.32; *p* = 0.026].

## Discussion

In this report on CPET in patients with ME/CFS, there are several notable findings. First, 71% of the patients with ME/CFS exhibited variability in ventilation consistent with dysfunctional breathing (42%), hyperventilation (32%), and/or an excessive ventilatory response to exercise (32%; elevated VE/VCO_2_ slope), compared to 32% of the sedentary controls (16% DB, 4% HV;32% elevated VE/VCO_2_). In the ME/CFS group, dysfunctional breathing was the most common ventilatory abnormality, followed by hyperventilation. The breathing patterns were mostly reproducible across consecutive studies. There was considerable overlap between DB and HV in the patients with ME/CFS, which was not observed in the sedentary controls. These findings suggest a potential new therapeutic target in the management of ME/CFS.

In this study, 32% of the patients with ME/CFS showed evidence of persistent hyperventilation, which is consistent with previous reports (3, 6). In addition, 50% of the patients with ME/CFS and hyperventilation also exhibited dysfunctional breathing. Dysfunctional breathing is rapid, irregular, shallow breathing and has considerable overlap with hyperventilation syndromes, with or without hypocapnia. Hyperventilation syndromes are frequently diagnosed using the Nijmegen Questionnaire, but more recently, CPET has been used to objectively measure respiration at rest and throughout exercise (4, 7). The combination of hyperventilation and dysfunctional breathing can result in a variety of symptoms, including dyspnea, fatigue, chest pain, palpitations, anxiety, and non-specific neurological symptoms such as tingling. The patients with hyperventilation at the start of exercise, whether acute or persistent, had a high frequency of dysfunctional breathing. These initial hyperventilation patterns can trigger dyspnea due to increased respiratory muscle workload. The identification of dysfunctional breathing and resting hypocapnia in this cohort is an important observation, as it may represent a target for treatment. Breathing retraining can be effective in relieving symptoms.

Both hyperventilation and dysfunctional breathing have been associated with autonomic dysfunction, that is, increased sympathetic and decreased parasympathetic tone leading to respiratory abnormalities, hypocapnia, and related symptoms. Vasoconstriction can result in decreased cerebral blood flow and symptoms of dizziness, fatigue, and mental fog. Tachypnea increases sympathetic tone and decreases sinus arrhythmia and heart rate variability (12, 13).

The effect of hypocapnia in producing symptoms is well known because over-breathing itself can often cause dizziness and an unwell feeling; this is probably due to hypocapnia-induced reduction in cerebral blood flow (12). Whether DB alone can cause symptoms is unclear because prior studies have not separated HV from DB. However, we did find more severe dyspnea when we combined HV with DB; increasing the sample size may allow us to clarify how each breathing disorder contributes to symptom severity. An important outcome of this study is the ability to stratify patients undergoing CPET into groups based on breathing patterns: normal breathing, dysfunctional breathing, hyperventilation, or a combination of both breathing disorders.

Even after excluding patients with persistent hyperventilation, nearly 40% of the patients with ME/CFS exhibited breathing patterns consistent with dysfunctional breathing (DB). DB is commonly found in individuals with asthma (7). Prolonged hypoxemia, metabolic abnormalities, and/or anxiety can trigger DB. In normal controls, the incidence of dysfunctional breathing is approximately 9% (10, 11). We previously reported that dysfunctional breathing occurred in 49% of patients with PASC (9). Following our initial report, other investigators have also reported a 29.4% incidence in patients with persistent dyspnea and ‘long COVID’ (26). We are planning future research to better understand the effect of DB on health.

The diagnosis of dysfunctional breathing remains challenging, as there is no consensus on the best method for its identification. There can be biomechanical, psychological, and physiologic mechanisms contributing to the abnormal breathing pattern. It may be an unconsciously learned habit of breathing resulting from psychological or physiologic stress. Questionnaire-based approaches, most commonly using the Nijmegen Questionnaire, manual assessment of respiratory motion, respiratory inductance plethysmography, and parameters measured during cardiopulmonary exercise testing have been used. We used the latter approach and focused on the oscillatory pattern of VE versus time and the variability in tidal volume, respiratory rate, and total ventilation. Others have advocated the use of VE/VCO_2_ equivalent at low-level exercise, such as a single point at 50 W > 35. In addition, some studies have used approximate entropy (27). The observation of a high frequency of disorganized breathing in patients with ME/CFS is important, as it provides a potential therapeutic target for this patient population via breathing retraining and pulmonary rehabilitation.

This study is limited by the lack of clear criteria for dysfunctional breathing. The use of a mouthpiece can be uncomfortable and result in hyperventilation and breathing irregularities at rest and during low-level exercise. A facemask might have been better tolerated by the patients. In addition, there are no biomarkers or diagnostic tests that clearly diagnose ME/CFS; similar to headache and psychiatric disorders, it is diagnosed based on a thorough medical history and the persistence of symptoms across time.

In conclusion, dysfunctional breathing and hyperventilation are observed frequently in patients with ME/CFS and are new therapeutic targets for these patients. Future studies with breathing retraining techniques should be considered to reduce symptoms and improve exercise performance.

## Data Availability

The raw data supporting the conclusions of this article will be made available by the authors without undue reservation.
